# Optical Properties and Local Structure Evolution during Crystallization of Ga_16_Sb_84_ Alloy

**DOI:** 10.1038/s41598-018-27972-3

**Published:** 2018-06-25

**Authors:** F. Dong, Y. R. Guo, C. Qiao, J. J. Wang, H. Shen, W. S. Su, Y. X. Zheng, R. J. Zhang, L. Y. Chen, S. Y. Wang, X. S. Miao, M. Xu

**Affiliations:** 1 Shanghai Ultra-Precision Optical Manufacturing Engineering Center and Department of Optical Science and Engineering, Fudan University, Shanghai, 200433 China; 2 National Taiwan Science Education Center, Taipei 11165, Taiwan and Department of Electro-Optical Engineering, National Taipei University of Technology, Taipei, 10608 Taiwan; 3 National Chip Implementation Center, Hsinchu, 30078 Taiwan; 4 Key Laboratory for Information Science of Electromagnetic Waves (MoE), Shanghai, 200433 China; 5 School of Optical & Electronic Information, Huazhong University of Science & Technology, Wuhan, 430074 China

## Abstract

Phase-change memory is one of the most promising candidates for future memory technologies. However, most of the phase-change memories are based on chalcogenides, while other families of materials for this purpose remain insufficiently studied. In this work, we investigate the optical properties and microstructure of Ga_16_Sb_84_ by an *in-situ* ellipsometer and X-ray diffraction. Our experimental results reveal that the Ga_16_Sb_84_ films exhibit a relatively high crystallization temperature of ~250 °C, excelling in long data retention. In addition, a large optical contrast exists between the amorphous and crystalline states, which may make it suitable for use in optical discs. Molecular dynamics simulations indicate that a unique local structure order in the amorphous and crystalline phases is responsible for the optical properties observed in the experiment. The similarity found in the short-range orders of the amorphous and crystalline phases is beneficial to better understanding the fast phase transition of phase-change memory.

## Introduction

Among all kinds of data storage media, phase-change memory is one of the most promising candidates for the next generation of nonvolatile memory applications due to its unique properties. For example, there are large differences in the optical and electrical properties of the amorphous and crystalline phases, which can then make data discernable^1–5^. The programming speed (i.e., the phase-change rate) can reach sub-nanoseconds, much faster than any other memory device^6^. Due to the exceptional performance, phase-change materials (PCMs) have been intensively studied since their invention in the 1960s^7^, and since then various PCMs have been discovered, such as Ge-Te^8–11^, Sb-Te^12–15^, and Ge-Sb-Te^16–21^.

Unlike traditional chalcogenides, the Ga-Sb alloy is a III-VA semiconductor which can potentially be used in PCMs. This material is reported to have large contrast in resistance and low difference in mass density between amorphous and crystalline phases. The crystallization temperature of stoichiometric GaSb is around 220 °C, showing better stability than amorphous Ge-Sb-Te^22–25^, with a phase-change speed of 10 ns in the recording process^26^. Normally, the crystalline phase of PCMs exhibits higher optical reflectivity and larger mass density than the amorphous phase. However, stoichiometric GaSb has switching properties opposite to regular PCMs, displaying reversed optical contrast and density change^27–29^. Moreover, the alloying of Sb in stoichiometric GaSb will produce a change in the crystallization temperature and electrical resistivity.

These unusual properties of Ga-Sb, such as mass density and optical contrast, are related to its atomic structure. Despite of some theoretical and experimental research on the Ga-Sb structure^27^, the underlying physics of the unusual Ga-Sb properties has not been fully understood. Furthermore, few studies of Ga-Sb alloys near the eutectic point have been carried out. Thus, in this work, optical properties of Ga_16_Sb_84_ were explored using an *in-situ* ellipsometer, while the changes in local structures from amorphous to crystalline phases were analyzed by *ab initio* molecular dynamics (AIMD) simulations. Specifically, we analyzed the optical and structural changes of Ga_16_Sb_84_ in the phase transition process. We found that the phase separation will reduce the optical contrast, and such a mechanism is detrimental to memory devices and should thus be avoided. Various methods are also used for the exploration of the crystalline procedure.

## Experiment and Simulation Method

### Experiment

The films were deposited at room temperature on Si (100) substrates using radio-frequency magnetron sputtering from a Ga_16_Sb_84_ target. The composition of the Ga-Sb films was confirmed by scanning electron microscope (SEM) and inductively coupled plasma-atomic emission spectrometry (ICP-AES). Scanning electron microscopy (SEM) techniques were used for the cross-sectional analyses of the as-deposited samples to determine thickness, which was about 1.2 um. The optical properties were measured with an *in-situ* ellipsometer with a temperature-controlled sample stage, which can be changed from 27 to 500 °C, over the spectra range of 300–800 nm at an incident angle of 70°. At each step, samples were annealed for 4 minutes and then measured for its optical properties directly by the variable-temperature spectroscopic ellipsometer at this temperature. As for the structure changes, the deposited samples were annealed at different temperatures for 15 min in the N_2_ environment, and then *ex-situ* characterized by X-ray diffraction (XRD).

### Simulation

The AIMD simulations were performed by the Vienna *ab initio* simulation package (VASP) code based on the density functional theory (DFT)^30,31^, with the projector-augmented wave (PAW) method and the Perdew-Burke-Ernzerhof generalized gradient approximation (GGA-PBE)^32,33^. A canonical ensemble with Nosé-Hoover thermostat was used to control the temperature^34,35^. Only the $\Gamma$ point was used to sample the Brillouin zone of the supercells. 32 Ga and 168 Sb atoms were randomly distributed in a cubic box initially, and then relaxed at a high temperature (1727 °C) for a long time to eliminate the memory effect from the initial configurations. Afterwards, the system was cooled down with a rate of 3.3 × 10^13^ °C /s to the temperatures of interest. For each sample temperature, the system is thermally equilibrated and the size of the simulation cell is adjusted to keep the external pressure zero. Afterwards, the system was relaxed for 4000 simulation steps to collect the structural information.

## Results and Discussion

**Figure 1:**
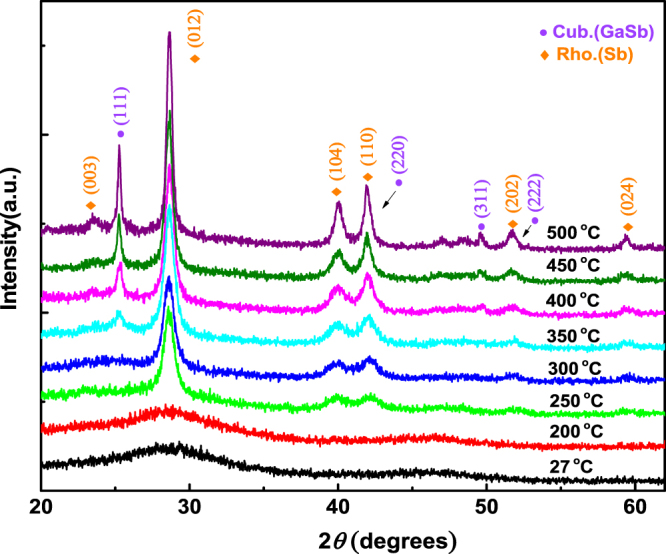
*Ex-situ* measured XRD patterns of Ga_16_Sb_84_ at different annealing temperatures.

To qualitatively investigate the overall structure transition of Ga_16_Sb_84_, the structures are measured by XRD, as shown in Fig. 1. Obviously, the amorphous phase is not crystallized below 200 °C. And it can be seen that several new peaks at about 28°, 40°, 42° and 59° appear as the temperature is increased to 250 °C, corresponding to the (012), (104), (110) and (024) of rhombohedral phase, respectively. At higher temperature of 350 °C, these peaks become sharper, and two other peaks at about 25° and 49° are found, corresponding to (111) and (311) of the cubic phase^22,23,36^. It should be pointed out that the peaks at 42° and 52° correspond to both of the cubic GaSb and rhombohedral Sb phases.

**Figure 2:**
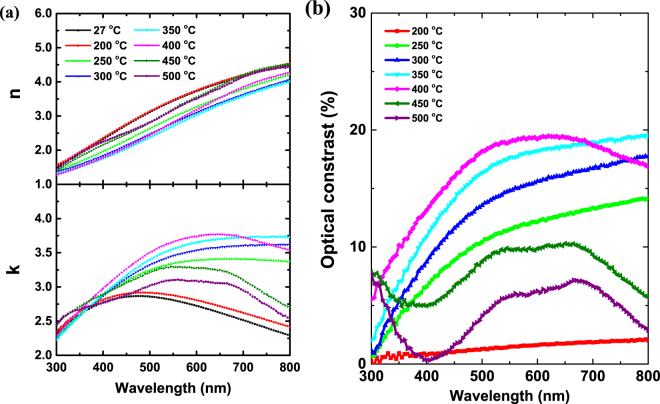
*In-situ* measured optical properties of Ga_16_Sb_84_ (**a**) complex refractive index, and (**b**) optical contrast.

Optical properties are relavant to the application of optical data storage. The refractive index *n* and the extinction coefficient *k* of Ga_16_Sb_84_ were measured by an *in-situ* ellipsometer at an incident angle of 70° with a heating rate of 2 °C/min. Figure 2(a) shows the changes of complex refractive index *n* and *k* with the temperature. The overall trend of optical properties at 200 °C-annealed sample is similar to that of the as-deposited one. As the temperature increases to 250 °C, the extinction coefficient *k* rises sharply in the 380–800 nm wavelength and refractive index *n* drops among all visible light regions. However, at higher temperature of 400 °C, *n* increases at about 480–800 nm and *k* exhibits a reduction at 690–800 nm. Such tendencies will probably persist with further increase of annealing temperature. Fig. 2(b) is the optical contrast of Ga_16_Sb_84_ at different temperatures, defined as^37^:

$${\rm{\Delta }}R/R=({R}_{a}-{R}_{{deposited}})/{R}_{{deposited}}$$

Where *R*_*a*_ is the optical reflectivity of samples, which are measured by variable-temperature spectroscopic ellipsometer at different temperatures and *R*_*deposited*_ is the reflectivity of room temperature. At 200 °C, the optical contrast raises a little with increasing wavelength. With the temperature increased to 250 °C, the appearance of rhombohedral phase lead to a sharp increase in optical contrast. When the temperature is over 400 °C, the optical contrast increases at 300–690 nm but reduces at other wavelengths, which is beneficial for the application of Blu-ray Disc (405 nm) but not as good for Compact Disc (780 nm). As the temperature turns to 450 °C, the overall trend of optical contrast decreases because of the occurred cubic phase. In PCMs, the large optical contrast between crystalline and amorphous phases is a basic requirement for memory applications, but the appearance of phase separation reduces the optical contrast, so it is harmful to phase-change storage when the annealing temperature above 350 °C.

The local structure is mainly responsible for the large property contrast of the PCMs^38^. To understand the structural change upon annealing, AIMD simulations were employed to explore the short-range order (SRO) of Ga_16_Sb_84_. In order to fully understand the evolution of the local structure changes related to the formation of the crystalline phases in the annealing process, various analytical methods were applied to quantitatively describe the structure of Ga_16_Sb_84_, including the pair correlation function (PCF), the bond angle distribution function, the atomic cluster alignment (ACA) method^39^ and local bond orientational order parameter^40^.

**Figure 3:**
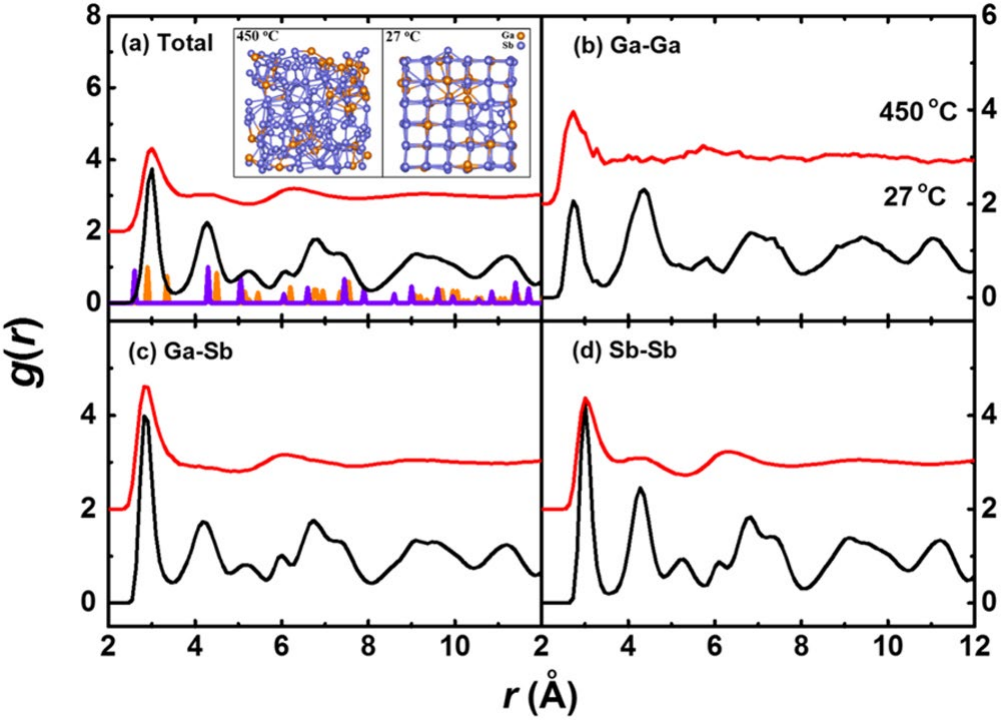
Total and partial PCFs of Ga_16_Sb_84_ at 450 and 27 °C, with the main peak positions of the perfect Sb and GaSb crystals represented by orange and purple bars, respectively. The inset is snapshots of Ga_16_Sb_84_ at these two temperatures.

Generally speaking, the solid state of the material is achieved by cooling from liquid state in the simulation and the local structure of liquid phase-change material is very similar to that of amorphous^41^. Our simulation attempts to find out the common characteristics of the local structure of amorphous and crystalline states and their role in the process of phase transition. The sample at 450 °C in the simulation is in a supercooled liquid of Ga_16_Sb_84_, having similar microstructure to amorphous state, while that at 27 °C represents the rhombohedral phase of the experiment. In order to study phase separation theoretically, a larger supercell is needed, but this is beyond the current computational power of AIMD,we just discuss the amorphous (at 450 °C) and crystalline (at 27 °C) phases in our simulations, revealing the evolution of short range order during the process from amorphous to rhombohedral phases. The inset in Fig. 3 indicated that at 450 °C little order of the atomic arrangement is observed. By further cooling to 27 °C, a distorted cubic phase emerges. PCF, which gives the probability of finding a particle in the distance *r* from a center atom, is used to investigate both phases, as plotted in Fig. 3. There are obvious differences between 450 °C and 27 °C in total and partial PCFs. In total PCF of Fig. 3(a), the height and amount of the peaks of 27 °C are much larger than those of 450 °C, demonstrating that the structures at low temperature are more well-defined than the liquid, consistent with snapshots. In spite of the difference between these two structures, the positions of first peaks in all PCFs move subtly, even though the temperature is changed. This implies that the distance of the nearest neighbors (i.e., the bond length) remains almost constant. Comparing the PCF of Ga_16_Sb_84_ to those of pure Sb and stoichiometric GaSb, we find that it more resembles Sb because of the same rhombohedral structure they share. Interestingly, the peaks of partial PCF in Ga-Sb and Sb-Sb are almost the same and the first peaks of the partial PCFs do not move with changing temperature.

**Figure 4:**
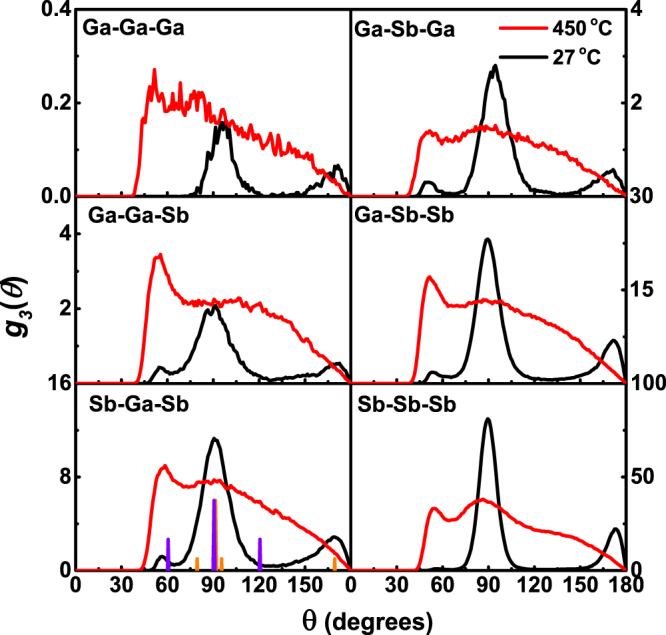
Bond angle distribution function at 450 and 27 °C, with the main peak angles of Sb and GaSb crystal represented by orange and purple vertical lines, respectively.

The bond angle distribution functions provide additional structural information other than PCF. Figure 4 shows that the crystalline Ga_16_Sb_84_ has become an octahedral-like structure because the peaks of bond angles are located at around 90° and 170°, a typical distorted cubic (rhombohedral) feature. Figure 4 also lists the angle distribution of perfect crystalline Sb, showing peaks at around 91° and 169° with a slight rhombohedral distortion. There are no obvious changes between Ga-centered angle (left) and Sb-centered one (right), confirming that Ga atoms may have a similar environment with Sb atoms in the solid solution, forming a distorted octahedron.

**Figure 5:**
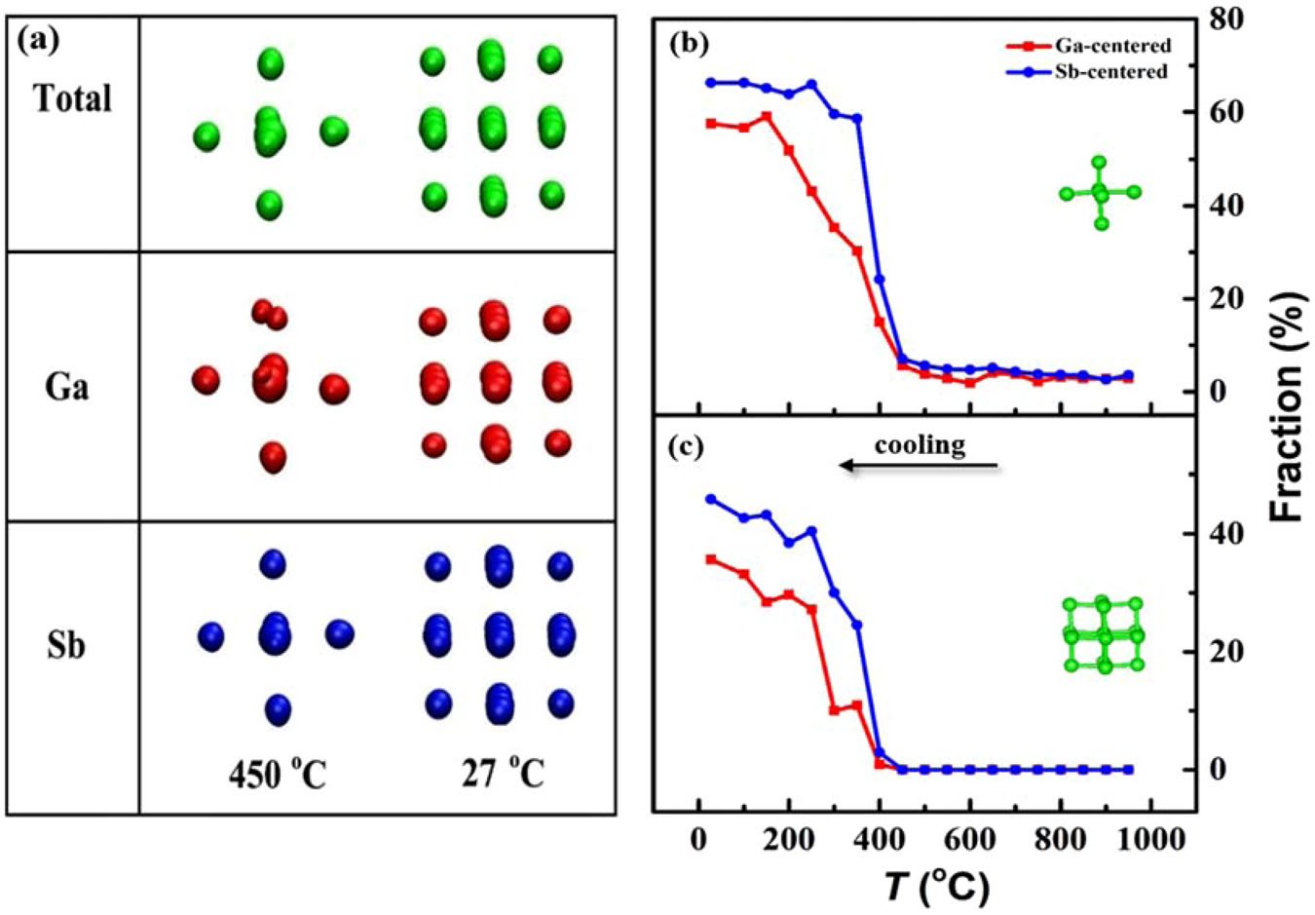
Analysis of cluster template alignment approach of Ga_16_Sb_84_ at different temperatures (**a**) collective alignment results for different centered clusters. Cluster-template alignments used by (**b**) 7 and (**c**) 19 atom templates (insets show the related template clusters).

The recently developed cluster template alignment approach in ACA^39^ methodology is utilized to provide a more comprehensive picture. Using this method, 2000 clusters with 21 atoms were randomly selected from a previous MD simulation and then rigidly rotated until the overall mean-square distances are minimized. Another Gaussian smearing scheme was adopted to smooth the three-dimensional atomic distribution to get a direct visualization of the local structural order, as displayed in Fig. 5(a). The images of Ga-centered (red), Sb-centered (blue) and overall clusters (green) resemble each other at 27 °C, showing a distorted cubic structure. The images at 450 °C are similar as well, with a slight separation for the Ga-centered pattern, which may stem from a slight distortion towards the tetrahedral-like local clusters around Ga. Nevertheless, the result demonstrates that Ga atoms have surroundings relatively similar to Sb in both phases. Compared with the ACA results of different centered clusters under different temperatures, the atoms at 450 °C are located in local clusters similar to those at 27 °C. Consequently, the amorphous short-range order is similar to that in the crystal, which remarkably increases the nucleation and growth rate and thus boosts the phase-change speed.

Template-cluster alignment in ACA was performed to obtain the quantitative results for short-range order. Templates were introduced for: body centered cubic (bcc, with 15 atoms), face center cubic (fcc, with 15 atoms), hexagonal close-packed (hcp, with 15 atoms), icosahedron (icos, with 15 atoms), zinc blende (zb, with 15 atoms) and rhombohedral clusters (distorted cubic with 7 and 19 atoms). It should be noted that there are not many differences between the 7 and 19 atom templates. The 7 atom template represents the short-range order, and the template with 19 atoms represents the medium-range order. Unlike some metallic glasses^42^ where icos is the dominating local motif, the amount of icos, bcc, fcc, hcp and zb in Ga_16_Sb_84_ is not detectable with the temperature change. The fraction of rhombohedral clusters for 7 and 19 atom templates for different centered clusters are illustrated in Fig. 5(b,c), respectively. At a high temperature over 450 °C, there are few rhombohedral short-range components. The percentage of the rhombohedral cluster, however, increases sharply when the temperature decreases to 400 °C. Further decrease of temperature increases the fraction of those 7 and 19 atom templates to ~65% and 45%. This confirms that the system is fully crystallized at 27 °C. This simulated cooling process resembles the crystallization in the experiment when the samples is annealed at 250 °C.

**Figure 6:**
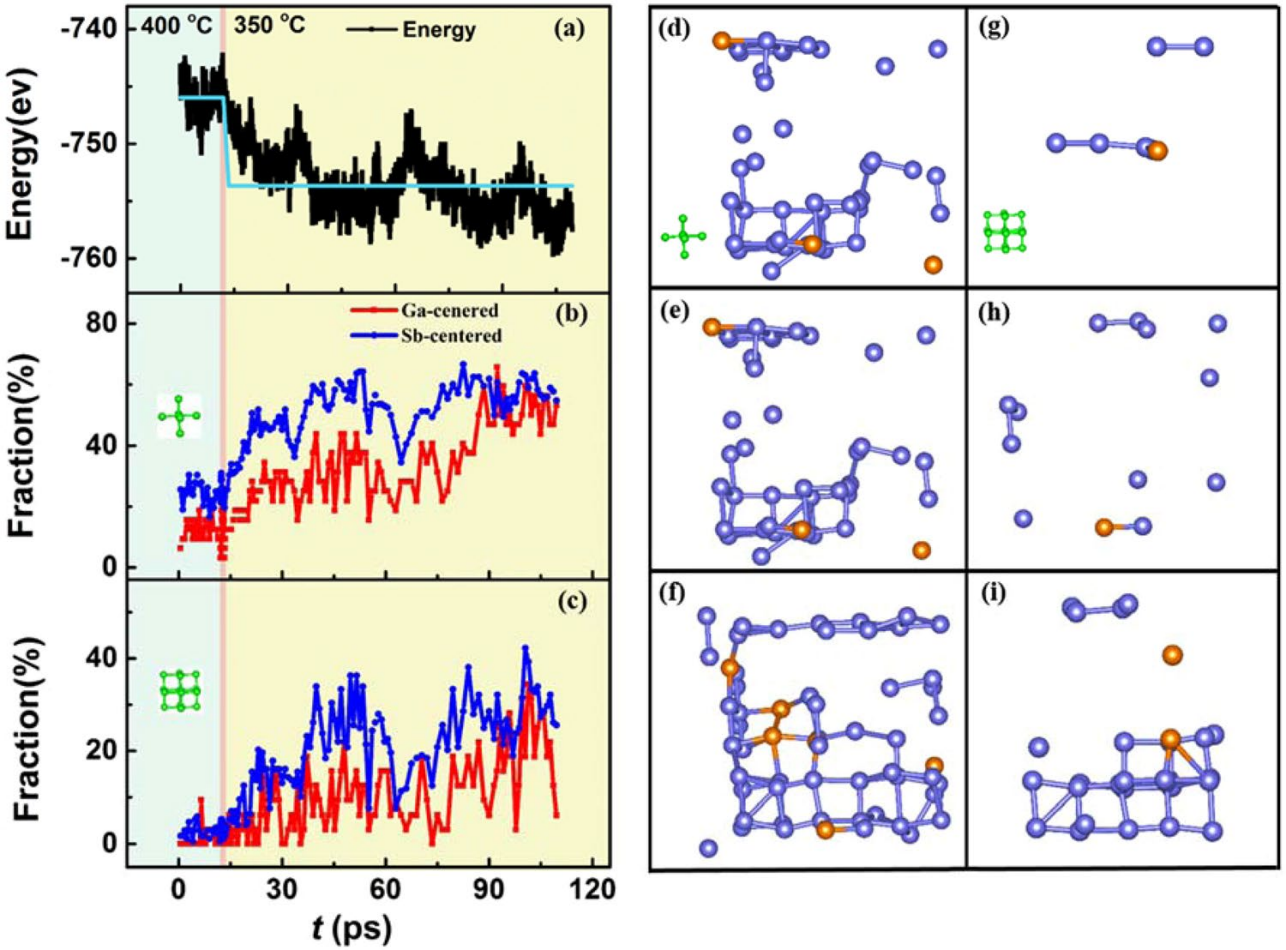
Nature of the crystallization process of Ga_16_Sb_84_. (**a**) Evolution of the total energy during crystallization. The light blue line is the average energy, relaxed at 400 and 350 °C. Fraction of individual cluster-template alignment in (**b**) 7 and (**c**) 19 atom templates. (**d**–**f**) Snapshots of central atoms selected from previous individual cluster-template alignment results for 7 atom template at the 12, 14.5 and 84 ps positions, respectively. (**g**–**i**) Corresponding snapshots as (**d**–**f**) for 19 atom template.

The nucleation process of the alloy upon crystallization is also studied, as illustrated in Fig. 6. The system is first relaxed at 400 °C for 12 ps (celadon background), and then cooled to 350 °C for 1.5 ps (reddish brown line). Finally, the system is stabilized for 108 ps (beige background). Figure 6(a) shows a clear decrease and increase of the energy release versus time, indicating a nucleation process. The individual cluster-template alignment fraction of the 7 and 19 atom templates are plotted in Fig. 6(b,c), respectively, as described by ACA methods. Interestingly, the fraction of the rhombohedral clusters increases with time, opposite from the trend in energy. This is because these crystal seeds have lower energy than the surrounding liquid phase, which is the driving force for crystal growth. Especially when the system overcomes the energy barrier and reaches a new low energy position, the fraction will increase precipitously. The detailed atomic arrangement is plotted in (**d**–**i**), matched with different templates. In the amorphous (supercooled liquid) phase, some rhombohedral clusters (crystal seeds) could emerge and even grow so as to decrease the energy of the system. Once the temperature decreases to 350 °C, the crystal seeds will grow fast, and the amorphous system swiftly transforms into a crystal.

**Figure 7:**
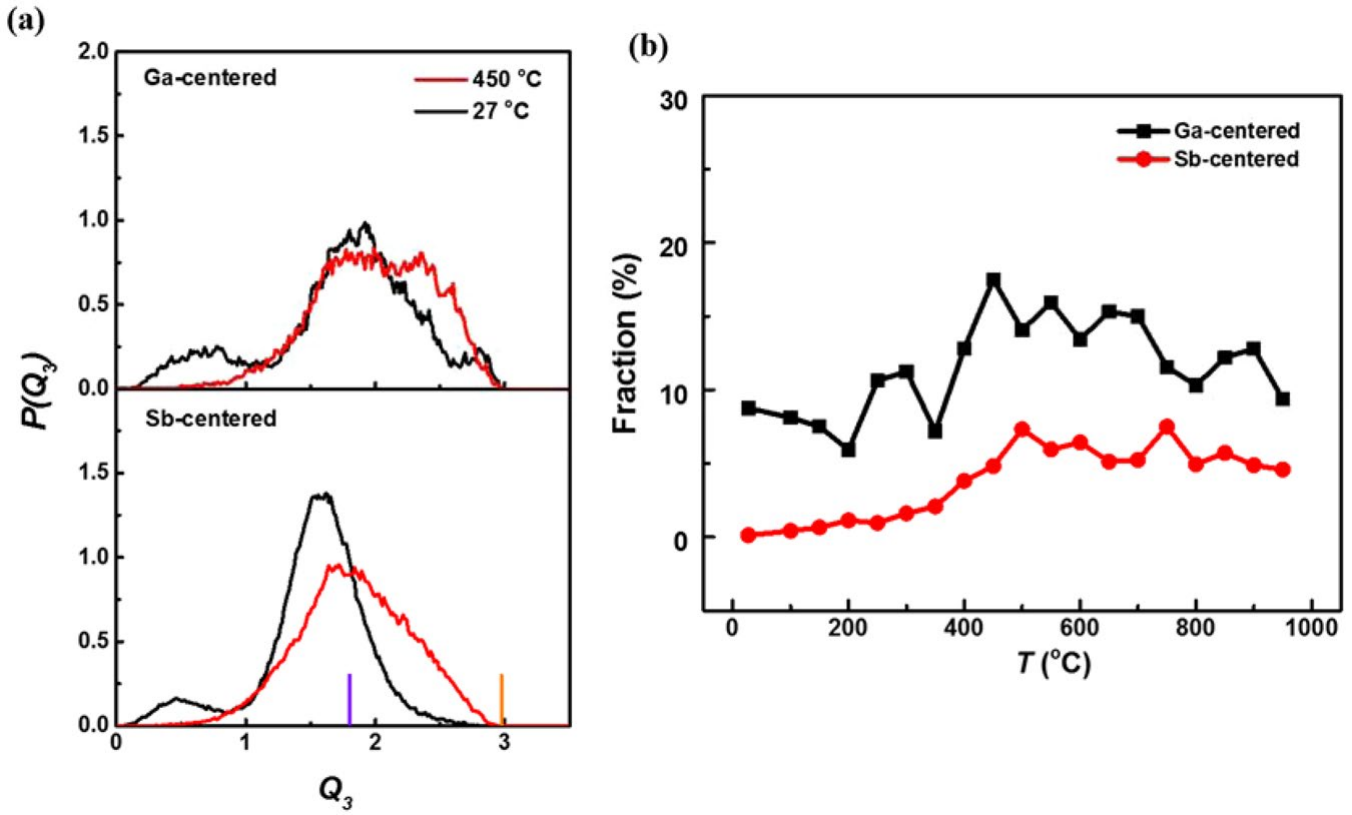
Analysis of the tetrahedral configuration of Ga_16_Sb_84_ at different temperatures. (**a**) Distribution of local bond orientational order parameter *Q*_3_, with the main peak position of Sb and GaSb crystals represented by orange and purple vertical lines, respectively. (**b**) The fraction of tetrahedrons with temperature by the ACA method.

Our simulation indicates that Ga_16_Sb_84_ at 27 °C forms a rhombohedral solid solution and no aggregation is detected. The process of phase separation of GaSb and Sb is far beyond the time/space scale of AIMD simulations. Thus, our simulations only focus on the Ga-Sb alloy from low-temperature annealing. The Steinhardt parameter *Q*_3_ is employed in the investigation, which can predict the GaSb phase in the alloy^40^. The calculated *Q*_3_ for the different temperatures of Ga_16_Sb_84_, and the corresponding values for Sb and stoichiometric GaSb structures (orange and purple vertical lines), are shown in Fig. 7. The peaks of total Ga- and Sb-centered clusters at 27 °C are sharper than those at higher temperature, implying the crystallization of the amorphous system. Moreover, compared with Sb-centered clusters, there is another broad peak near 2.8 for Ga-centered clusters. Therefore, this suggests some crystal seeds of GaSb may exist in the system. In order to obtain the fraction of tetrahedrons (local clusters in the zinc blende GaSb), different kinds of clusters are selected from Ga- and Sb- centered clusters at 4000 steps by the ACA method. It is clear that at high temperature the fraction of tetrahedrons is enhanced with decreasing temperature, as depicted in Fig. 7(b). Once the temperature is lower than 400 °C, the number of tetrahedrons decreases, likely due to the crystallization of the alloy. However, the fraction is enhanced again at a temperature lower than 200 °C, which implies that the GaSb phase may indeed appear if long-time and large-scale simulation is performed. It is of interest to note that the most favored clusters are Ga-Sb_4_, and its fraction is much higher (over 60%) than that of Ga-Ga_1_Sb_3_ and Ga-Ga_2_Sb_2_, indicating that Ga atoms tend to form tetrahedrons with Sb atoms.

## Conclusions

In summary, XRD and an *in-situ* ellipsometer were utilized to study the changes in optical and microstructural properties of the Ga_16_Sb_84_ alloy from its amorphous to crystalline phases. Results indicate crystallization temperature of ~250 °C, corresponding to the abrupt structural and optical property changes. High temperature annealing will lead to phase separation of Ga_16_Sb_84_, and two-phase separation will decrease of optical contrast, which is not conducive to optical phase change storage. We show that the local structure difference in different states is responsible for this difference in the optical properties observed in experiment. The evolution of the local structure from amorphous to crystalline phase is also studied using AIMD simulations, and the fraction of rhombohedral clusters increases to ~65% and 45% by the use of template-cluster alignment method for 7 and 19 atom templates. A few tetrahedrons may exist which represent the crystal seeds of zinc blende GaSb. Moreover, Ga and Sb atoms are located in similar chemical environments derived from the partial PCF and bond angle distribution in crystalline Ga_16_Sb_84_ alloys, indicating that they form mixed solid solutions. Such a Ga_16_Sb_84_ alloy has a similar structural motif in the amorphous and crystalline phases, responsible for fast crystallization. Our results provide useful knowledge about the structure and optical properties of Ga-Sb materials upon crystallization and have implications for the design of new phase change memories.
